# Effects of Elastic Band Based Plyometric Exercise on Explosive Muscular Performance and Change of Direction Abilities of Male Team Handball Players

**DOI:** 10.3389/fphys.2020.604983

**Published:** 2020-12-16

**Authors:** Ghaith Aloui, Souhail Hermassi, Mehrez Hammami, Yosser Cherni, Nawel Gaamouri, Roy J. Shephard, Roland van den Tillaar, Mohamed Souhaiel Chelly

**Affiliations:** ^1^Research Unit (UR17JS01) «Sport Performance, Health and Society», Higher Institute of Sport and Physical Education of Ksar Saîd, University of “La Manouba”, Tunis, Tunisia; ^2^Sport Science Program, College of Arts and Sciences, Qatar University, Doha, Qatar; ^3^Higher Institute of Sport and Physical Education of Ksar Said, University of “La Manouba”, Tunis, Tunisia; ^4^Faculty of Kinesiology and Physical Education, University of Toronto, Toronto, ON, Canada; ^5^Department of Sport Sciences and Physical Education, Nord University, Levanger, Norway; ^6^Sports Performance Research Institute New Zealand, Auckland University of Technology, Auckland, New Zealand

**Keywords:** stretch-shortening cycle, peak power, sprinting, team sports, turning

## Abstract

This study examined the effects of incorporating 8 weeks of bi-weekly lower limb elastic band based loaded plyometric training into the in-season regimen of junior handball players. Participants were assigned between control (*n* = 15, age: 18.1 ± 0.5 years, body mass: 73.7 ± 13.9 kg, height: 1.82 ± 0.06 m, body fat: 14.4 ± 6.0%) and experimental groups (*n* = 14, age: 17.7 ± 0.3 years, body mass: 76.8 ± 10.7 kg, height: 1.83 ± 0.04 m, body fat: 13.4 ± 3.8%). Measures obtained before and after the intervention included a cycle ergometer force-velocity test, squat and countermovement jump characteristics, sprints times, repeated change of direction and change of direction tests (COD), a 1-RM half-back squat, and anthropometric estimates of limb volumes. Gains in the experimental group relative to controls included absolute muscle power (W) (Δ 23.1%; *p* < 0.05; ES = 0.565), relative muscle power (W.kg^–1^) (Δ 22.1%; *p* < 0.05; ES = 0.573), sprint times over 5 and 30 m (Δ−8.7%; *p* < 0.01; ES = 0.921 and Δ−7.2%; *p* < 0.05; ES = 0.573, respectively), COD times (Δ−9.2%; *p* < 0.05; ES = 0.561) and all repeated COD parameters except the fatigue index. However, a significant improvement by time interaction was observed in both groups on some anthropometric parameters (leg muscle volume and surface section thigh max), 1-RM half- back squat and vertical jump performance. We conclude that bi-weekly elastic band-loaded plyometric training improves the ability to sprint, COD and repeated COD relative to regular training, and thus it can be recommended to young male team handball players as a new method of plyometric training to improve important elements of their physical performance.

## Introduction

Team handball is considered a change of direction sport (Wagner et al., 2014) and during matches, rapid changes of direction (COD) actions are considered among the activities most frequently performed (Karcher and Buchheit, 2014). In offensive and defensive actions throughout a match, the player can perform many COD actions and can travel an average of 14 to upwards of 711 meters side steps at moderate to high intensity (Povoas et al., 2012; Karcher and Buchheit, 2014).

Plyometric training (Hermassi et al., 2014; Hammami et al., 2016) and strength training (Hermassi et al., 2011, 2017) are two possible options for the enhancement of physical performance in team athletes. The former elicits the stretch-shortening cycle, inducing gains predominantly in sprinting and repeated change of direction ability (Hammami et al., 2016). Despite suggestions that strength training might hamper the development of speed and ability to change direction (Hammami et al., 2019), some reports have now demonstrated significant gains in the speed and agility of team athletes from programs that combine isometric and plyometric training (Garcia-Pinillos et al., 2014; Latorre Roman et al., 2018).

Several studies have also reported that plyometric training combined with variable resistance provided by a Smith machine (Lyttle et al., 1996; McBride et al., 2002), a weighted vest (Markovic et al., 2013; Negra et al., 2019), weighted discs and bar (Coratella et al., 2018), or handheld dumbbells (Rosas et al., 2016; Kobal et al., 2017) are all effective methods of improving measures of athletic performance. Negra et al. (2019) showed significant increases in the ability of young soccer players to change direction, sprint, and make horizontal and vertical jumps after 8 weeks of a bi-weekly plyometric program wearing a weighted vest, and Kobal et al. (2017) noted significant gains in sprinting and vertical jumping of young male soccer players after 6 weeks of bi-weekly of plyometric training using handheld dumbbells.

However, there remains an interest in simple and inexpensive but effective ways that could be coupled with plyometric training in this fashion. One such option is elastic band training. It provides a safe and progressive method of activating all muscle groups, applicable not only to athletes, but also to sedentary people and injured patients of all ages, even at home. Because they stimulate different aspects of muscular contraction, a combination of plyometric and elastic band training might indeed maximize the response. Thus it was thought of interest to examine empirically the response to a combination of plyometric and elastic band strength conditioning, comparing the resulting gains in performance to control data, and to the gains observed when using either plyometric (Hermassi et al., 2014) or elastic (Aloui et al., 2019) training alone.

Therefore, the present investigation evaluated the effects upon the performance-related abilities of junior male handball players of replacing a part of their normal in-season training by a combined elastic band and plyometric training program. The hypothesis tested was that replacing a part of the regular in-season training by a bi-weekly 8 week intervention of this type would enhance both measures of lower limb muscular strength and power such as jump height, and indicators of speed and agility such as change of direction scores relative to control players who maintained their standard in-season regimen, with gains in each of these domains being of at least the order anticipated if either elastic band or plyometric training had been introduced without the second training modality.

## Materials and Methods

### Participants

The 29 participants were drawn from all playing positions on a single male handball team competing in the first national division. Their mean experience of competition was 6.3 ± 0.7 years. Before acceptance into the study, they were examined by the team physician, with a focus upon orthopedic and other conditions that might preclude plyometric training. Participants were randomly assigned between an experimental group (*n* = 14, age: 17.7 ± 0.3 years, body mass: 76.8 ± 10.7 kg, height: 1.83 ± 0.04 m, body fat: 13.4 ± 3.8%) and a control group (*n* = 15, age: 18.1 ± 0.5 years, body mass: 73.7 ± 13.9 kg, height: 1.82 ± 0.06 m, body fat: 14.4 ± 6.0%). A Student’s *t*-test showed no significant initial inter-group differences of anthropometric characteristics between the two groups (*p* ≤ 0.05).

All participants had already achieved a good general physical condition at the beginning of the season, having already completed a preliminary 8 week period with 5–6 training sessions per week. During the first five of these weeks, they had followed a resistance training program aimed at improving muscular volume by moderate loads [60–70% 1 repetition maximum (RM)] and muscle strength [by heavy loads (80–95% 1RM)]. The remaining 3 weeks had been devoted to improving muscular power with light to moderate loads (40–60% 1RM), with participation in friendly matches every weekend. Participants continued to participate in five sessions per week of training when the championship season had begun.

### Experimental Design

Participants avoided any physical training other than that associated with handball practice throughout the study. The standard routine for both experimental and control groups consisted of five training sessions per week (∼90 min each session), plus a competitive game each weekend. Physical training was undertaken twice a week; the first session aimed at developing anaerobic fitness through half squats, overhead lunges, countermovement and squat jumps, push-ups, and pull-ups), supplemented by moderately loaded strength exercises for both lower and upper limbs (40–60% 1RM exercises such as bench presses, pull-overs, and half back squats). The second session of the week aimed at developing aerobic fitness through high intensity interval training and small-sided games (Dello Iacono et al., 2016). The remaining sessions sought to develop tactical technical skills (60% of session time) and physical abilities (40% of session time). All participants also engaged in weekly 60 min school physical education sessions (Table 1).

**TABLE 1:** Details of general training routine during the week performed by both control and experimental groups over the 8 week intervention.

*Days*	Mondays	Tuesdays	Wednesdays	Thursdays	Fridays	Saturdays
Objectives	*Rest*	Integrated aerobic training Defensive tactics training	Maximum power aerobic training. Technical drills	Power anaerobic training. Tactical training based on counter-attacks	Technical training. Offensive and defensive tactics training	Official games

All procedures were approved by the University Institutional Review Committee for the ethical human experimentation, according to current national laws and regulations. All participants (and their guardians, in the case of minors) read and signed informed consent documents in accordance with University Institutional Review Committee guidelines, and they were assured that they could withdraw from the trial without penalty at any time. Two familiarization sessions were held 2 weeks before definitive testing, which began 2 months into the competitive season.

#### Training Program

**FIGURE 1:**
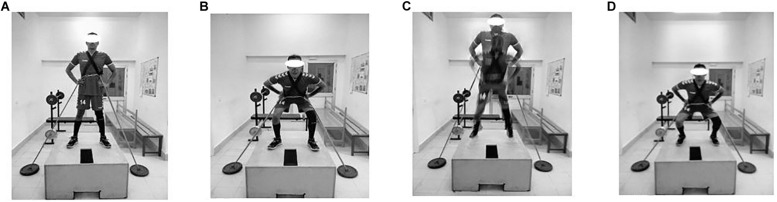
Phases of countermovement jump. **(A)** Starting position, **(B)** Drop countermovement phase, **(C)** Flight phase, and **(D)** Reption phase.

**FIGURE 2:**
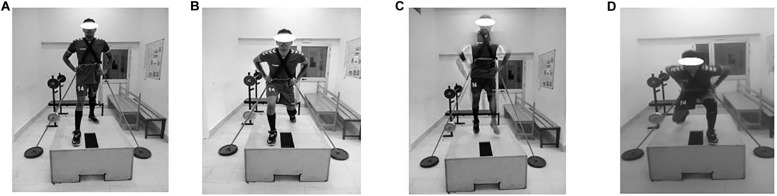
Phases of split squat jump. **(A)** Starting position, **(B)** Drop countermovement phase, **(C)** Flight phase, and **(D)** Reption phase.

Every Tuesday and Thursday during the 8 week intervention, the experimental group replaced the technical-tactical part of their standard regimen with elastic band-loaded plyometric training (Table 2). The latex bands (Thera-Bands^®^; Hygenic Corporation; Akron, Ohio, United States) were of differing elasticity: silver (Super Heavy) and gold (Maximum Heavy). Participants were instructed to perform all exercises with maximal effort. The program included countermovement (Figure 1) and split squat jumps (Figure 2). Training sessions began with a 15 min warm-up and lasted for a further 20 min. The standardized warm-up included trunk rotation, internal and external rotary movements of the hip, squat, split squat, squat pulses, lateral displacement, front-to-back displacement, skipping with and without changes of direction, knee elevation, heels to buttocks, countermovement jumping, split squat, 15–20 m sprinting with and without change of direction. 40–50 push-ups with both hands on the ground and 8–10 free-medicine-balls throws with both hands. 6 silver elastic bands, 8 silver elastic bands, 6 gold elastic bands, and 6 gold plus 2 silver elastic bands were used, respectively for the first and second, third and fourth, fifth and sixth and seventh to twelfth weeks. The initial length of the elastic band was 60 cm; it was extended to 125% (approximately 145 cm) when the participant was in half-squat position. Exercises were selected based on the muscle groups involved in handball.

**TABLE 2:** Details of elastic band-based plyometric training program performed by the experimental group over the 8 week intervention.

Exercises	Session 1	Session 2	Session 3	Session 4	Session 5	Session 6	Session 7	Session 8
		
	With 6 silver elastic bands at 125% elongation (41.4 kg)				With 8 silver elastic bands at 125% elongation (55.2 kg)			
Countermovement jump	5 × 6	5 × 6	6 × 6	6 × 6	5 × 6	5 × 6	6 × 6	6 × 6
Split-squat jump	5 × 6	5 × 6	6 × 6	6 × 6	5 × 6	5 × 6	6 × 6	6 × 6

	**Session 9**	**Session 10**	**Session 11**	**Session 12**	**Session 13**	**Session 14**	**Session 15**	**Session 16**
		
	**With 6 gold elastic bands at 125% elongation (67.2 kg)**				**With 6 gold plus 2 silver elastic bands at 125% elongation (81 kg)**			

Countermovement jump	5 × 6	5 × 6	6 × 6	6 × 6	5 × 6	5 × 6	6 × 6	6 × 6
Split-squat jump	5 × 6	5 × 6	6 × 6	6 × 6	5 × 6	5 × 6	6 × 6	6 × 6

*(sets× reps).*

#### Countermovement Jump

The subject stood on a wooden box (height 50 cm, length 120 cm, width 90 cm), with a strap running laterally from the hip to the ground. With the legs held slightly apart, the knees and hips were flexed to 90 degrees, and the participant then jumped as high as possible by extending the ankles, knees, and hips. On landing, the trunk was inclined slightly forward, the head was aligned with the spine and the back was held rigid. The impact was absorbed by lowering the body into a squat, and the next jump was initiated immediately, until the required number of repetitions had been performed (Figure 1).

#### Split Squat Jump

The participant stood on the wooden box, as above. With the legs held slightly apart, one step was taken forward into a split stance, quickly dropping into a split squat with the leading thigh parallel to the floor and the rear thigh perpendicular to the floor. The participant then jumped upwards, and while in the air, quickly switched the legs forward and backward by performing a scissor-kick, keeping the toes pulled to the shins to prepare for the next landing. As soon as the feet landed, with the trunk inclined slightly forward and the back held rigid, the impact was absorbed by lowering the body into a split squat. The next jump was initiated immediately and jumping continued for the prescribed number of repetitions (Figure 2).

The intervention began 1 week after baseline testing. Loading began at a resistance of 41.4 kg, increasing by 12–13 kg every four sessions to reach a final value of 81 kg. The number of sets was also increased from 5 to 6 after each two sessions at a given level of resistance. Six repetitions per set were performed throughout training. No injuries were incurred over the 16 workouts.

#### Testing Schedule

Initial and final test measurements were performed at the same time of day (17:00–19:00 p.m.), under approximately the same environmental conditions (temperature: 16–19°C), at least 3 days after the most recent competition, and 5–9 days after the last session of plyometric and elastic band training, with 2 days of rest between test sessions. A standardized battery of warm-up procedures preceded each measurement procedure. On the first test day, anthropometric assessments were followed by squat and countermovement jumps, and then the force–velocity test. On the second day, sprinting was followed by the one repetition (1RM) half-back squat, and on the third day, the change of direction test (T-half test) was followed by the repeated change of direction test.

#### Day 1

##### Anthropometry

Standard equations predicted the percentage of body fat from measurements of biceps, triceps, subscapular, and suprailiac skinfolds (Womersley and Durnin, 1973).

%Bodyfat=alog(Σ4folds)-b

where ΣS is the sum of the 4 skinfolds (in mm), and a and b are sex and age dependent constants.

##### Leg Muscle Volume

Measurements of the circumferences at the maximal level of the calf just above the ankle and skinfolds on the back and each side of the calf plus leg length (from the trochanter major to the lateral malleolus) were added to data for the thigh to calculate the leg muscle volume.

##### Mean Cross Sectional Area of the Thigh (CSA)

The mean thigh CSA was calculated from maximal and mid-thigh circumferences; considering the latter as a circle, its radius R was calculated as:

Radius (R) = Circumference (C)/2π

The radius for the muscular component of the mid-thigh (r) was estimated by allowing for the double thickness of anterior and posterior skin folds:

r = R – [(mid-thigh anterior skin fold + mid-thigh posterior skin fold)/4]

The thigh CSA was then equal to π⋅ r^2^ (cm^2^).

##### Squat and Countermovement Jumps

Characteristics of the squat and counter-movement jumps (jump height, maximal force before take-off, maximal velocity before take-off and the average power of the jump) were evaluated using a force platform (Quattro Jump, version 1.0.9.2, Copyright 2002–2007^®^, Acquisition Rate 500 Hz, Kistler Instruments AG, Winterthur, Switzerland). The maximal force before take-off was identified as the peak force recorded, at the end or at the beginning of the pushing phase during the force-time curve of the squat and countermovement jumps respectively. The time between take-off and contact after flight was then used in the equation of uniform acceleration:

$$h=\frac{gt_{f}^{2}}{8}$$

where:

h: jump height

g: acceleration of gravity (9.81 m/s_2_)

t_*f*_: flight time

Participants began the squat jump at a knee angle of 90°, avoiding any downward movement, and they performed a vertical jump by pushing upwards, keeping their legs straight throughout. The countermovement began from an upright position, participants performing a rapid downward movement to a knee angle of 90° and simultaneously beginning to push-off. One minute of rest was allowed between the three trials of each test, the highest jump being used in subsequent analyses.

##### Force–Velocity Test

Force–velocity measurements for the lower limbs were performed on a standard Monark cycle ergometer (model 894 E, Monark Exercise AB, Vansbro, Sweden) as detailed elsewhere (Chelly et al., 2010). In brief, the instantaneous maximal pedaling velocity during a 7 s all-out sprint was used to calculate the maximal anaerobic power for each braking force, and the participant was judged to have reached peak power (Wpeak) if an additional load induced a decrease in power output. Measured parameters included Wpeak, maximal braking force (F_0_) and maximal pedaling velocity (V_0_). The relationship between braking force F and velocity V can be expressed by the equation:

$$V=b-\mathrm{aF or V}=V_{0}-V_{0}\;F/F_{0}\;\mathrm{or}=V_{0}(1-F/F_{0})$$

where V_0_ is the intercept on the velocity axis, i.e., the theoretical maximal velocity for a braking force of zero, and F_0_ is the intercept on the force axis, i.e., the theoretical maximal braking force corresponding to a velocity of zero. After 10 min of standardized warm-up, the formal test began at a braking force equal to 1.5% of the participant’s body mass. After each 5 min recovery interval, the braking force was increased in sequence to 2.5, 5, 7.5, 9, and 11.5% of the individual’s body mass (Chelly et al., 2010).

#### Day 2

##### Sprint Performance (30 m)

The 30 m sprint began with a standardized warm-up. Participants started from a standing position, with the front foot 0.2 m behind the photocell beam. They ran 40 m to ensure that the participant did not drop their speed at 30 m, with times at 5 and 30 m being recorded by paired photocells (Microgate, Bolzano, Italy). Three trials were separated by 6–8 min of recovery and the fastest performance was considered in statistical analysis.

##### One Repetition Maximum of Back Half-Squat

The back half-squat test was used to estimate maximal leg extensor strength. The 1RM was calculated as the maximum weight that the participant could lift over the whole range of motion from 90° knee flexion. Participants maintained an upright position. The bar, supported on the shoulders, was grasped firmly with both hands. The knees were bent to 90° and the upright position was then regained, with the legs fully extended (Aloui et al., 2019). The 1RM was approximated during familiarization sessions. Warm-up consisted of five repetitions at loads of 40–60% of the perceived maximum. To measure definitive 1RM values, the barbell was loaded with free weights to an initial 90% of the pretest 1RM (Aloui et al., 2019). After two successful repetitions at the pretest 1RM and a 3 min recovery interval, a further 10 kg load was added (Aloui et al., 2019). If the second repetition could not be completed at the new loading, this value was accepted as the individual’s 1RM.

#### Day 3

##### Change of Direction test (T-Half Test)

**FIGURE 3:**
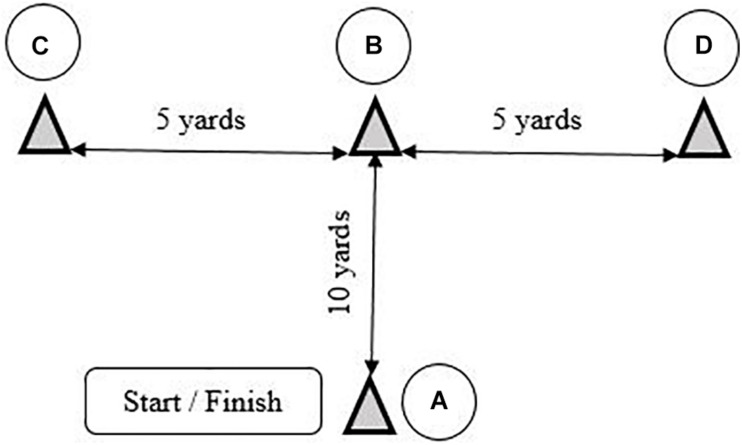
**(A–D)** T-half test design.

The T-half test (Sassi et al., 2009) was performed using the same protocol as the *T*-test, (Figure 3). Participants began with both feet behind the starting line A. After sprinting forward to cone B and touching its base with the right hand, they shuffled left to cone C, touching its base with the left hand. They then shuffled to the right to cone D, touching its base with the right hand, subsequently shuffling back to cone B and touching its base. Finally, they ran backward to line A. Anyone who crossed one foot in front of the other failed to touch the base of a cone, and/or failed to face forward throughout had to repeat the test. The faster of two final trials as timed by infra-red sensors (Microgate, Bolzano, Italy) was used for statistical analysis.

##### Repeated Change of Direction Test (Repeated COD)

**FIGURE 4:**
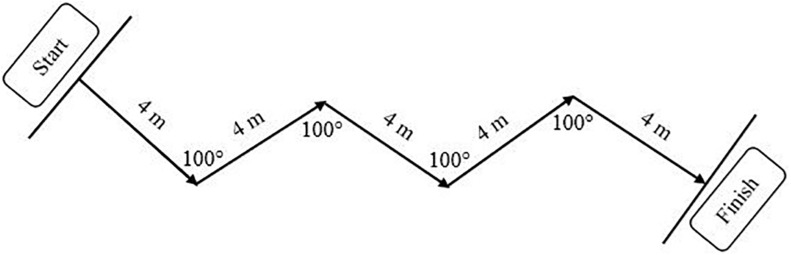
Repeated change-of-direction test design.

The repeated COD test comprised 6 × 20 m sprints (Figure 4), each beginning from a standing position 0.2 m behind the initial infra-red sensor (Microgate, Bolzano, Italy), with 25 s active recovery intervals. Four 100° turns were made at 4 m intervals, with participants jogging slowly back to the starting line during the active recovery phase. Data collected included the fastest time in a single trial (repeated COD best), the average time for the 6 × 20 m sprints (repeated COD mean), the total time for the 6 repetitions (repeated COD total), and the repeated COD decrement calculated as (Glaister et al., 2008):

Repeated COD fatigue index = 100 × (total sprint time ÷ ideal sprint time)) – 100

Where:

Total sprint = Sum of times from all sprints

Ideal sprint = The number of sprints × fastest sprint time

### Statistical Analyses

Statistical analyses were carried out using the SPSS 20 program for Windows (SPSS, Armonk, NY: IBM Corp, United States). The normality of data was tested using the Shapiro-Wilk test and results showed that all values were normally distributed. Means and SDs were calculated, and independent *t*-tests examined between-group differences at baseline. Paired sample *t*-tests evaluated within-group pre-to-post performance changes. Training-related effects were assessed by 2-way analyses of variance (ANOVA) with repeated measures (group × time). Where significant *F*-values were observed, Tukey’s *post hoc* procedure was applied to locate pair-wise differences. An analysis of covariance (ANCOVA) was also run for variables where there were baseline inter-group differences. Effect sizes were determined by converting partial eta-squared to Cohen’s d (Cohen, 1988); values were classified as small (0.00 ≤ d ≤ 0.49), medium (0.50 ≤ d ≤ 0.79), and large (d ≥ 0.80). The reliability of measures was assessed using intra-class correlation coefficients and the coefficients of variation over consecutive pairs of intra-participant trials. All measures of change of direction ability, vertical jumping and sprinting showed an ICC > 0.80 and a CV < 5%. Statistical significance was set at *p* ≤ 0.05, whether a positive or a negative difference was seen (i.e., a 2-tailed test was adopted).

## Results

Intra-class correlation coefficients, confidence intervals, and coefficients of variation assessing the reliability for track speeds, change of direction, and vertical jump tests are summarized in Table 3. The majority of parameters showed no initial inter-group differences. Training-related effects were evaluated using 2-way analyses of variance with repeated measures. However, one measure (Wpeak/surface section 1/2 thigh) showed some initial inter-group difference, and an ANCOVA was then applied.

**TABLE 3:** Inter-class correlation coefficient (ICC, 95% confidence limits) and coefficient of variation (CV), showing acceptable reliability for measures of track running velocity, change of direction, squat jump and countermovement jump tests.

	ICC	95%CI	CV
**Track running times**			
5 m(s)	0.93	0.85–0.97	1.7
30 m(s)	0.98	0.96–0.99	1.3
**Change of direction test**			
T half test (s)	0.98	0.96–0.99	1.1
**Squat jump**			
Power (W)	0.99	0.99–1.00	2.2
Power (W.kg^–1^)	0.98	0.97–0.99	2.5
Velocity (m.s^–1^)	0.97	0.94–0.99	2.5
Force (N)	0.99	0.99–1.00	1.8
Height (m)	0.95	0.90–0.98	3.8
**Countermovement jump**			
Power (W)	0.97	0.94–0.99	3.7
Power (W.kg^–1^)	0.99	0.97–0.99	2.5
Velocity (m.s^–1^)	0.88	0.69–0.94	3.1
Force (N)	0.99	0.97–0.99	2.7
Height (m)	0.98	0.96–0.99	2.4

### Effect of Training on Anthropometric Measures

There were no significant group × time interactions for anthropometric measures (Table 4). However, paired *t*-tests indicated a significant improvement in all muscle volumes for the experimental group and the control group also showed gains in leg muscle volume and maximal surface section of the thigh.

**TABLE 4:** Comparison of muscle volume of lower limbs and% body fat between experimental and control groups before and after the 8 weeks intervention.

	Experimental (*n* = 14)					Control (*n* = 15)					ANOVA (group× time)	
	Pre	Post	% change	*P*-value	ES	Pre	Post	% change	*p*-value	ES	*P-*value	ES
Leg muscle volume (L)	8.8 ± 2.0	9.4 ± 1.9	7.3 ± 8.2	0.008*	0.302	10.1 ± 2.6	10.5 ± 2.6	4.0 ± 4.1	0.001*	0.141	0.859	0.063 (small)
Thigh muscle volume (L)	5.8 ± 1.5	6.3 ± 1.4	9.3 ± 10.1	0.003*	0.315	6.6 ± 1.8	6.8 ± 2.2	1.2 ± 18.7	0.418	0.088	0.754	0.089 (small)
Surface Section 1/2 thigh (cm^2^)	172 ± 47	182 ± 45	6.6 ± 7.3	0.006*	0.221	202 ± 46	204 ± 48	1.2 ± 4.5	0.264	0.052	0.051	0.275 (small)
Surface section thigh max (cm^2^)	209 ± 48	221 ± 45	6.5 ± 7.7	0.011*	0.254	234 ± 70	242 ± 69	4.3 ± 5.2	0.004*	0.126	0.922	0.063 (small)
Body fat%	13.4 ± 3.8	13.2 ± 3.5	−0.5 ± 8.4	0.562	0.045	14.4 ± 6.0	14.6 ± 5.8	0.17 ± 5.0	0.260	0.029	0.899	0.063 (small)

A 2-way analysis of variance (group× time) assessed the statistical significance of training-related effects. *Indicates a significant difference from pre-post test for this group on a p < 0.05 level.

### Effect of Training on Power Performance

The only significant (group × time interactions) favoring the experimental group compared to the control group, for the force–velocity test were in terms of absolute muscle power (W) (Δ 23.1%; *p* < 0.05; ES = 0.565), relative muscle power (W.kg^–1^) (Δ 22.1%; *p* < 0.05; ES = 0.573), and power was expressed per unit of surface section (1/2 thigh or thigh max [W.cm^2^−1]) (Δ 15.9%; *t*-test *p* < 0.01; ES = 2.184) (Table 5).

**TABLE 5:** Force-velocity test data, 1RM strength, sprint times, and change of direction performance for experimental and control groups before and after the 8 weeks intervention.

	Experimental (*n* = 14)					Control (*n* = 15)					ANOVA (group× time)		ANCOVA (group× time)	
	Pre	Post	% change	*p*-value	ES	Pre	Post	% change	*p*-value	ES	*p*-value	ES	*p*-value	ES
**Force–velocity test**														
Wpeak (W)	627 ± 106	770 ± 118	23.1 ± 4.2	0.001*	1.275	619 ± 86	651 ± 94	5.2 ± 3.6	0.001	0.355	0.042†	0.565 (medium)		
Wpeak (W.kg^–1^)	8.5 ± 1.0	10.4 ± 1.2	22.1 ± 1.2	0.001*	1.725	8.4 ± 1.9	8.6 ± 1.7	3.0 ± 3.3	0.012*	0.118	0.040†	0.573 (medium)		
Wpeak (W/total leg.muscle.volume)	72.7 ± 9.7	83.5 ± 10.5	15.3 ± 7.3	0.001*	1.075	64.2 ± 15.4	64.5 ± 13.2	1.3 ± 5.6	0.786	0.020	0.114	0.439 (small)		
Wpeak (W/surface section 1/2 thigh)	3.8 ± 0.6	4.4 ± 0.7	15.9 ± 6.7	0.001*	0.898	3.2 ± 0.7	3.3 ± 0.7	4.2 ± 6.1	0.036*	0.162			0.001	2.184 (large)
V0 (rpm)	179 ± 16	196 ± 18	9.6 ± 3.8	0.001*	0.985	179 ± 16	185 ± 18	3.4 ± 4.7	0.013	0.361	0.231	0.326 (small)		
F0 (N)	144 ± 24	149 ± 27	3.4 ± 3.8	0.006*	0.195	148 ± 25	149 ± 28	0.7 ± 5.9	0.577	0.050	0.790	0.063 (small)		
**1RM strength**														
1RM half-back squat (kg)	123 ± 15	133 ± 16	7.6 ± 1.1	0.001*	0.590	122 ± 10	125 ± 11	2.5 ± 2.1	0.001*	0.298	0.438	0.238 (small)		
**Sprint**														
5 m (s)	1.16 ± 0.05	1.06 ± 0.04	−8.7 ± 2.4	0.001*	2.271	1.17 ± 0.07	1.16 ± 0.07	−0.89 ± 2.2	0.120	0.152	0.005†	0.921 (large)		
30 m (s)	4.82 ± 0.20	4.47 ± 0.19	−7.2 ± 1.2	0.001*	1.774	4.83 ± 0.37	4.81 ± 0.35	−0.38 ± 3.7	0.640	0.063	0.039†	0.573 (medium)		
**Change of direction**														
T-half test (s)	6.19 ± 0.34	5.62 ± 0.31	−9.2 ± 0.54	0.001*	1.739	6.19 ± 0.20	6.01 ± 0.27	−3.7 ± 1.4	0.001*	0.759	0.044†	0.561 (medium)		

V0 present the maximal pedaling velocities. F0 represent the maximal braking forces. Wpeak represent the maximal power output for lower limbs. A 2-way analysis of variance (group× time) assessed the statistical significance of training-related effects. *Indicates a significant difference from pre-post test for this group on a p < 0.05 level. ^†^Indicates a significant difference from pre-post test between the groups on a p < 0.05 level.

### Effect of Training on Maximum Muscular Strength Performance

In terms of maximal strength performance, the back half-squat test showed no significant group × time interactions, although paired *t*-tests indicated performance improvements in groups (experimental Δ 7.6%; *p* < 0.01; ES = 0.593; control Δ 2.5%; *p* < 0.01; ES = 0.298) (Table 5).

### Effect of Training on Sprint Performance

The present results showed significant intervention effects (group × time interaction), in the experimental group compared to the control, on the sprint performance (Δ−8.7%; *p* < 0.01; ES = 0.921 and Δ−7.2%; *p* < 0.05; ES = 0.573 over distances of 5 and 30 m, respectively) (Table 5).

### Effect of Training on Change of Direction Ability

In terms of change of direction ability, the T-half test showed an intervention effect favoring the experimental group compared to the control group (Δ−9.2%; *p* = 0.044; ES = 759) (Table 5).

### Effect of Training on Repeated Change of Direction Ability

For the ability to perform repeated change of direction, the repeated COD test showed group × time interactions for most parameters (repeated COD best, mean and total), with time decreases of Δ−7.6% (*p* < 0.05; ES = 0.552), Δ−7.1% (*p* < 0.05; ES = 0.552), and Δ−7.1% (*p* < 0.05; ES = 0.557), respectively (Table 6).

**TABLE 6:** Repeated change of direction and vertical jump test performances in experimental and control groups before and after 8 week intervention.

	Experimental (*n* = 14)					Control (*n* = 15)					ANOVA (group× time)	
	Pre	Post	% change	*p*-value	ES	Pre	Post	% change	*p*-value	ES	*p*-value	ES
**Repeated change of direction parameters**												
Fastest Time (s)	6.92 ± 0.35	6.44 ± 0.20	−7.6 ± 4.2	0.001*	1.722	6.94 ± 0.40	6.84 ± 0.42	−1.5 ± 2.6	0.042*	0.259	0.047†	0.552 (medium)
Mean Time (s)	7.12 ± 0.33	6.61 ± 0.21	−7.1 ± 3.0	0.001*	1.843	7.13 ± 0.41	7.00 ± 0.43	−1.7 ± 3.1	0.049*	0.296	0.047†	0.552 (medium)
Fatigue Index (s)	2.9 ± 1.1	2.7 ± 1.7	−1.1 ± 61.3	0.681	0.120	2.70 ± 1.20	2.47 ± 1.3	−1.7 ± 46.7	0.303	0.178	0.940	0.557 (medium)
Total Time (s)	42.7 ± 2.0	39.7 ± 1.3	−7.1 ± 3.0	0.001*	1.843	42.8 ± 2.5	42.03 ± 2.6	−1.7 ± 3.1	0.047*	0.296	0.046†	0.557 (medium)
**Counter-movement jump**												
Power (W)	1,874 ± 424	1,957 ± 412	5.0 ± 7.9	0.025*	0.198	1,842 ± 360	1,874 ± 350	2.0 ± 5.6	0.268	0.100	0.237	0.102 (small)
Power (W.kg^–1^)	25.3. ± 2.9	26.2 ± 3.4	3.7 ± 6.3	0.049*	0.298	24.3 ± 4.4	24.6 ± 4.1	1.6 ± 4.8	0.310	0.077	0.257	0.094 (small)
Velocity (m.s^–1^)	2.54 ± 0.15	2.64 ± 0.17	3.6 ± 4.2	0.006*	0.586	2.51 ± 0.20	2.53 ± 0.20	0.7 ± 2.5	0.335	0.081	0.026†	0.347 (small)
Force (N)	1,504 ± 219	1,533 ± 202	2.2 ± 5.1	0.213	0.139	1,496 ± 266	1,512 ± 294	0.9 ± 5.7	0.490	0.059	0.697	0.001 (small)
Height (cm)	39.8 ± 3.4	43.4 ± 3.9	9.1 ± 3.7	0.001*	0.987	38.8 ± 5.6	40.4 ± 6.2	4.0 ± 3.2	0.001*	0.267	0.001†	0.767 (medium)
**Squat jump**												
Power (W)	1,529 ± 410	1,649 ± 390	8.9 ± 8.9	0.001*	0.299	1,576 ± 415	1,619 ± 420	2.9 ± 3.5	0.007*	0.103	0.014†	0.417 (small)
Power (W.kg^–1^)	20.5 ± 2.5	21.9 ± 2.4	7.1 ± 8.6	0.006*	0.556	20.5 ± 3.5	20.9 ± 3.5	2.0 ± 2.8	0.010*	0.114	0.031†	0.326 (small)
Velocity (m.s^–1^)	2.45 ± 0.19	2.56 ± 0.19	4.6 ± 3.9	0.001*	0.577	2.44 ± 0.20	2.47 ± 0.21	0.91 ± 2.1	0.013*	0.146	0.007†	0.499 (small)
Force (N)	1,499 ± 256	1,607 ± 259	7.6 ± 5.4	0.001*	0.421	1,540 ± 273	1,569 ± 280	1.9 ± 2.1	0.004*	0.105	0.001†	0.787 (medium)
Height (cm)	37.8 ± 4.2	41.3 ± 4.2	9.4 ± 3.4	0.001*	0.832	36.1 ± 5.2	37.9 ± 5.6	4.8 ± 2.1	0.001*	0.325	<0.001†	0.963 (large)

Fastest time present the best time in a single trial. Mean time present the average time for the 6 sprints. Fatigue index presented the index of decrement of the 6 sprints. Total time presents the sum of times of the 6 sprints. A 2-way analysis of variance (group× time) assessed the statistical significance of training-related effects. *Indicates a significant difference from pre-post test for this group on a p < 0.05 level. ^†^Indicates a significant difference from pre-post test between the groups on a p < 0.05 level.

### Effect of Training on Jump Performance

Vertical jumping was unaffected by the intervention, although both groups showed significant improvements in height (experimental group Δ 9.4%; *p* < 0.01; ES = 0.832 on squat jump; Δ 9.1%; *p* < 0.01; ES = 0.987 on countermovement jump; control group Δ 4.8%; *p* < 0.01; ES = 0.325 and on squat jump; Δ 4%; *p* < 0.01; ES = 0.267 on countermovement jump) (Table 6).

## Discussion

The aim of the present study was to test the effects of bi-weekly lower limb elastic band based loaded plyometric training into the in-season regimen on strength and muscle power of the lower limbs, muscle volume, sprinting, change of direction and repeated changes of direction ability, and jumping in elite adolescent handball players. We have observed that all quality and physical ability measures have been improved for the experimental group except muscular strength and vertical jumping which were not improved relative to data for individuals following the standard training protocol.

### Muscle Power Performance

Handball is a very complex sport where success depends on several basic abilities, including the ability to develop power in repeated explosive movements (Povoas et al., 2012). The present force–velocity data indicated significant intervention effects on absolute muscle power (W) (Table 5). These results corroborate those of Markovic et al. (2013) who compared the effectiveness of 8 weeks of bi-weekly vest-loaded or unloaded plyometric training on the maximal and average power of lower limb muscles in male physical education students; they observed gains in maximal and average muscle power during jump squat exercises [8.4% (ES = 0.67)] and [18.6% (ES = 0.99), respectively] with increases in the maximal and average muscle power developed during countermovement jumps [7.5% (ES = 0.67)] and [7.9% (ES = 0.57), respectively], after the loaded plyometric training. These researchers observed approximately the same increase in average muscle power during jump squat exercise after unloaded plyometric training, although there was then no significant improvement of the maximal muscle power developed during the same exercise. In additional, Lyttle et al. (1996) observed significant improvements in the absolute power (9%; *p* < 0.05) after 6 weeks of bi-weekly loaded plyometric training in adult male athletes. In contrast, Kobal et al. (2017) showed trivial improvements in maximum power relative to body mass following loaded [0.7% (ES < 0.2)] and unloaded [1.7% (ES = 0.2)] plyometric training in elite young male soccer players. In the short time of the current study, improved stretch-reflex (Avela et al., 1996) and higher eccentric overload during plyometric training with additional loads (Coratella et al., 2018), may have contributed to greater improvements in muscle activation and greater improvements in the efficiency of the stretch-shorten cycle (Negra et al., 2019). The difference in between studies especially at the level where the percentage improvement in performance could be explained by differences in the initial physical level of the participants, in the age group of participants, in the intervention period during the sports season, and in the plyometric training model used (loaded or unloaded).

### Anthropometric Measures and Maximum Muscular Strength Performance

In the present study, we saw no increases of muscle volumes, implying that the observed increases in muscle power reflected neuronal adaptations (Behm and Sale, 1993; Behm, 1995). Increases of muscular strength would give a clear advantage in maintaining handball specific movements (Gorostiaga et al., 2005). Our results showed a significant effect over time in both groups with no significant group × time interactions (Table 5). These results disagree with McBride et al. (2002) who reported improvements in 1RMS (8.3 and 9.9% for Smith-machine loaded jump squat training at 30 and 80% of 1RMS, respectively; *p* ≤ 0.05). Likewise, Coratella et al. (2018) showed moderate effects on the 1RM performance of recreational soccer players [12.7% (ES = 0.73)] from 8 weeks of plyometric training loaded by weight discs and bar, and smaller effects [7.4% (ES = 0.4)] after unloaded plyometric training. Contradictions in these results could be explained by differences in training modalities, frequency (e.g., number of sessions by week), duration (e.g., number of weeks of training), intensity (e.g., drop-height), direction of jumping (e.g., horizontal, vertical) and the number of limbs involved (i.e., unilateral, bilateral jumping).

### Jump Performance

Jump height is vital to handball success (Wagner et al., 2014). However, the present results showed no significant intervention effects (group × time interaction) on SJ or CMJ height, although both groups showed significant interaction effects between time, and the experimental group showed greater improvement than the control as shown by the effect size (Table 6). Despite the absence of significant benefit in the experimental group, the increase in the vertical performance of both groups was similar to that seen in other studies of loaded (Rosas et al., 2016; Kobal et al., 2017; Negra et al., 2019) and unloaded (Hermassi et al., 2014) plyometric training in young and adult athletes.

The lack of response of jump performance in the present study could reflect an insufficient intensity (number of contacts or weight of the additional load) or volume (number of training sessions) of training, or the fact that the players were initially in good physical condition and our limited choice of jump tests (jump without arm motion, only vertical jump). Several authors have mentioned that the initial low level of physical condition may explain the magnitude and speed of the jump performance gains that they observed (Fatouros et al., 2000; Martínez-López et al., 2012).

### Speed Performance

Perhaps because of the substantial increases in muscle power, the present results also showed significant intervention effects, in the experimental group compared to the control, on the sprint performance over distances of 5 and 30 m (Table 5). Our results are in agreement with those observed by other studies after loaded plyometric training programs in young and adult male soccer players (Coratella et al., 2018; Negra et al., 2019). In contrast, previous studies did not observe a significant improvement in sprint performance after loaded plyometrics training program (McBride et al., 2002; Kobal et al., 2017). A high eccentric overload (Coratella et al., 2018), improving the degree of muscular coordination (Fatouros et al., 2000; Martínez-López et al., 2012), and an improved stretch reflex (Avela et al., 1996) may have contributed to a greater improvement in muscle activation, stretch-shortening cycle efficiency, and stiffness of muscular-tendinous tissue due to the loaded plyometrics (Negra et al., 2019).

### Change of Direction Ability

Handball involves multidirectional changes of direction (Massuca et al., 2014) in response to unpredictable stimuli (Karcher and Buchheit, 2014). Previous studies have reported that the ability to change direction is one of the most important factors of successful play in handball (Wagner et al., 2014). To our knowledge, this is the first study to investigate loaded plyometrics in the context of rapid changes of direction. The present results showed significant intervention effects favoring the experimental group compared to the control group (Table 5). Our findings are in accordance with previous studies which have already reported positive effects of loaded and unloaded plyometric training programs, in young and adult athletes (McBride et al., 2002; Coratella et al., 2018; Negra et al., 2019). Indeed, Sheppard and Young (2006) suggested that loaded plyometric training improved the eccentric strength of the thigh muscles, and consequently enhanced performance during the deceleration phase of changes in direction of movement. There is likely a change in neural drive to the agonists, favoring a quick switch between deceleration and acceleration, increased inter-and intra-muscular coordination, and/or increased proprioception (Hakkinen et al., 1985; Impellizzeri et al., 2008; Markovic and Mikulic, 2010).

### Repeated Change of Direction Ability

Significant improvements were seen in all repeated COD parameters except the fatigue index favoring the experimental group compared to the control group (Table 6). The absence of significant change in the fatigue index parameter may reflect its poor reproducibility (Impellizzeri et al., 2008). The current findings are in accordance with previous studies which have already reported positive effects of loaded (Coratella et al., 2018; Negra et al., 2019) and unloaded (Rosas et al., 2016) plyometric training programs, in young and adult athletes. These results are in agreement with Hammami et al. (2016), who studied the effects of unloaded plyometric training in young male soccer players; they, also, reported improvements of around 4.1% in all repeated COD parameters except the fatigue index. Likewise, Hermassi et al. (2014) noted that in tests of repeated-sprint ability (RSA) without change of direction, elite handball players showed improvement in all RSA parameters in response to unloaded plyometrics.

## Limitations

Our observations to date are primarily applicable to junior handball players at a specific level of competition, and there is a need to extend observations to cover female players, other age groups, and other skill levels. Further, despite efforts to match participants across groups, there were some pre-test differences in anthropometric parameters, although we did adjust for these by covariance techniques. Further, there remains a need to compare the gains of performance we have seen with improvements of actual play on the handball court. Finally, since vertical jumping and muscle strength are vital qualities for handball players, there is a need to explore whether an increase of training intensity or volume could make the novel regimen that we have evaluated effective in these domains. While taking due account of these limitations, the ability to improve sprinting and change of direction, by elastic band-loaded plyometric offers a tempting approach to conditioning that is cheaper than the use of Smith machines, with wide-ranging implications for athletes and coaches alike.

## Conclusion

This controlled study shows that elite junior male handball players who are already participating in a demanding training schedule and consider themselves to be well-trained can make further substantial gains in some handball-related performance measures if they replace a part of their standard regimen by an in-season 8 week bi-weekly program of elastic band-loaded plyometric training. Handball is a very complex activity that depends on several factors, including anthropometrics and physical abilities, with fast, dynamic movements and body contact present throughout the match in offensive and defensive situations. Our results have shown that elastic band loaded plyometric training is very useful to meet the physical needs of this game. Nevertheless, it remains to be demonstrated by further testing that the suggested modification of training not only improves scores on performance tests, but also enhances match performance.

## Data Availability Statement

The raw data supporting the conclusions of this article will be made available by the authors, without undue reservation, to any qualified researcher.

## Ethics Statement

The studies involving human participants were reviewed and approved by the Manouba University Institutional Review Committee. The participants provided their written informed consent to engage in this study. Written informed consent was also obtained from the individual(s) for the publication of any potentially identifiable images or data included in this article.

## Author Contributions

MSC and GA contributed to the formal analysis and contributed to the project administration. GA, SH, MH, YC, and NG investigated the study and performed the methodology. MSC and SH supervised the study. GA, SH, MH, and MSC wrote the original draft of the manuscript. RS, SH, RVT, and MSC wrote, reviewed, and edited the manuscript.

## Conflict of Interest

The authors declare that the research was conducted in the absence of any commercial or financial relationships that could be construed as a potential conflict of interest.
